# Beyond MyD88 and TRIF Pathways in Toll-Like Receptor Signaling

**DOI:** 10.3389/fimmu.2014.00070

**Published:** 2014-02-24

**Authors:** Vincent Piras, Kumar Selvarajoo

**Affiliations:** ^1^Institute for Advanced Biosciences, Keio University, Tsuruoka, Japan; ^2^Systems Biology Program, Graduate School of Media and Governance, Keio University, Fujisawa, Japan

**Keywords:** Toll-like receptors, macrophages, innate immunity, Pearson correlation, gene expression

**Figure 1:**
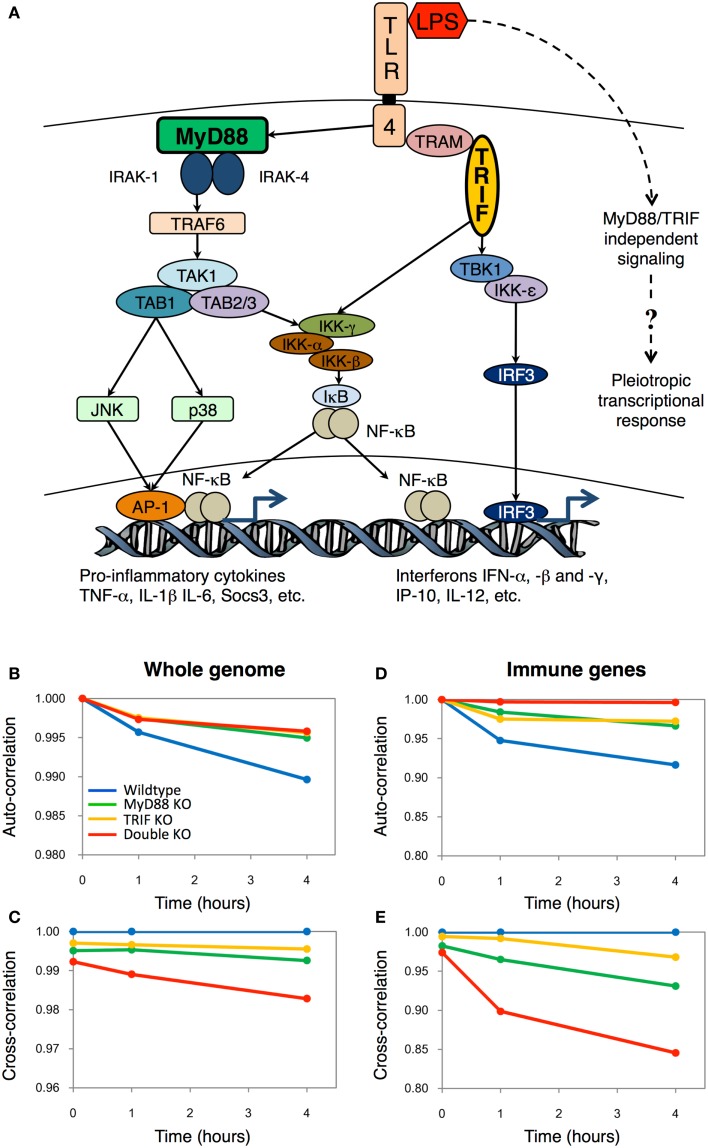
**The Toll-like receptor-4 mediated transcriptional responses in wildtype and mutant macrophages**. **(A)** Schematic topology showing the major players of the Toll-like receptor-4 signaling. The dotted line indicates the hypothetic pathways activating the pleiotropic transcriptional response independently from the TLR4 signaling. Auto-correlations and cross-correlations for whole genome **(B,C)** and 157 important immune genes **(D,E)** in LPS-stimulated murine macrophages. *x*-axis represents time (in hours after LPS stimulation), and *y*-axis represents the correlation coefficient (with *t* = 0 h for auto-correlations, and between KO genotypes and wildtype for cross-correlations). Figure adapted from Ref. (2).

The Toll-like receptors (TLRs), 13 types known to-date, are a major class of transmembrane proteins of the mammalian innate immune system (1). They are known to detect diverse pathogen-associated molecular patterns of microorganisms, and trigger specialized sets of signal transduction cascades that neutralize any danger posed to the host by the intruders. The major adaptors that bind to the intracellular domain of TLR to activate the proinflammatory response are the myeloid differentiation primary response (MyD) 88 and TIR-domain-containing adapter-inducing interferon-β (TRIF). Together, MyD88 and TRIF lead to the expression of numerous cytokines, such as TNF-α, IL-1β, IL-6, IP-10, IFN-γ, etc., through transcriptional factors NF-κβ, AP-1, and IRF-3 activation (Figure 1A).

In a September 2013 issue of the journal *Science*, Hagar et al. (3) and Kayagaki et al. (4) reported a major discovery in the TLR4 based innate immune response. For the first time, both research groups demonstrated the ability of Gram-negative bacteria, through lipopolysaccharides (LPS), to trigger a novel proinflammatory response independent of the TLR4. Collectively, they showed that caspase-11, which plays a pivotal role in shaping inflammasome, is activated intracellularly without the need for TLR4. This finding is a key advancement in the TLR field after the discovery of MyD88 and TRIF over a decade ago (5, 6). So, is this the beginning of recognizing a MyD88/TRIF-independent response?

To share our thoughts, here we summarize our previous work on high throughput LPS response in macrophages (2). We investigated the genome-wide response of LPS-stimulated murine macrophages in four experimental conditions [wildtype, MyD88 knock-out (KO), TRIF KO, and MyD88/TRIF Double KO (DKO)] at three time points (0, 1, and 4 h). Instead of the commonly used approach of discarding gene expressions below an arbitrarily chosen threshold-cutoff, which highly limits the spectrum of genes analyzed, we undertook a novel approach of analyzing the entire 22,690 ORFs from the Affymetrix-based microarray dataset. We do appreciate the fact that microarray or even the recently developed RNA-Seq datasets are prone to a large degree of error or biases, especially for the lowly expressed genes. However, our goal was not to specifically identify individual novel genes expressed in all four conditions. Instead, we examined the global collective behaviors of the LPS-induced innate immune response (7).

We mainly adopted the statistical Pearson correlation analysis, which is widely used to observe global patterns in complex systems such as the weather (8), stock markets (9), and cosmology (10). In essence, when two samples containing high-dimensional (such as microarray) data are compared, the correlation analyses provide a measure of deviation from unity as a source of difference between the samples. In our case, the Pearson correlation coefficient shows the compressed (averaged) information of the genome-wide response. We developed a scheme to compare the correlation coefficients between (i) the same genotype at different times (e.g., wildtype 0 h vs. wildtype 1 h, called *auto-correlation*) and, (ii) the same time point with different genotypes (e.g., wildtype 1 h vs. MyD88 KO 1 h, called *cross-correlation*) (Figures 1B,C).

From the correlation plots, we surprisingly observed that DKO auto-correlations were similar to single KOs on the temporal scale (Figure 1B). In short, this is an indication that LPS is able to invoke gradual intracellular response independent of the key adaptor molecules MyD88 and TRIF, as seen by the monotonic deviation of correlation coefficient from unity. Nevertheless, the cross-correlations showed that DKO response, compared with wildtype, is the least similar (Figure 1C). This result indicated that although DKO showed gene expression response to LPS, its effect is the least among the four genotypes.

To confirm whether DKO induces genome response, we, subsequently, compared correlation coefficients of whole genome with an ensemble comprising of 157 well-known proinflammatory genes (Figures 1D,E). Notably, for the selected group of proinflammatory genes, the auto-correlation for DKO was almost unchanged with time, indicating their nullified response in DKO, consistent with other studies (11). Altogether, these results indicated the presence of unknown pathways, independent of MyD88 and TRIF, to activate novel gene expressions in DKO (Figure 1A, dotted line). Although we had pointed out a few biological processes not specifically related to immunity using the Gene Ontology database, we could not experimentally verify the specific DKO or TLR4-independent response of LPS at that time. Nevertheless, today, we are delighted of the recent findings of Hagar et al. and Kayagaki et al. Their work not only brings a fresh perspective to TLR4 research, but also indirectly supports the utility of using simple Pearson statistical analysis to uncover novel regulatory response from genome-wide expression dataset.
